# Machine Learning Techniques to Predict Timeliness of Care among Lung Cancer Patients

**DOI:** 10.3390/healthcare11202756

**Published:** 2023-10-18

**Authors:** Arul Earnest, Getayeneh Antehunegn Tesema, Robert G. Stirling

**Affiliations:** 1 School of Public Health and Preventive Medicine, Monash University, Melbourne, VIC 3004, Australia; getayeneh.tesema@monash.edu; 2 Department of Respiratory Medicine, Alfred Health, Melbourne, VIC 3004, Australia; r.stirling@alfred.org.au; 3 Central Clinical School, Faculty of Medicine, Nursing and Health Sciences, Monash University, Melbourne, VIC 3168, Australia

**Keywords:** machine learning, lung cancer, timeliness of care, socio-economic disadvantage

## Abstract

Delays in the assessment, management, and treatment of lung cancer patients may adversely impact prognosis and survival. This study is the first to use machine learning techniques to predict the quality and timeliness of care among lung cancer patients, utilising data from the Victorian Lung Cancer Registry (VLCR) between 2011 and 2022, in Victoria, Australia. Predictor variables included demographic, clinical, hospital, and geographical socio-economic indices. Machine learning methods such as random forests, k-nearest neighbour, neural networks, and support vector machines were implemented and evaluated using 20% out-of-sample cross validations via the area under the curve (AUC). Optimal model parameters were selected based on 10-fold cross validation. There were 11,602 patients included in the analysis. Evaluated quality indicators included, primarily, overall proportion achieving “time from referral date to diagnosis date ≤ 28 days” and proportion achieving “time from diagnosis date to first treatment date (any intent) ≤ 14 days”. Results showed that the support vector machine learning methods performed well, followed by nearest neighbour, based on out-of-sample AUCs of 0.89 (in-sample = 0.99) and 0.85 (in-sample = 0.99) for the first indicator, respectively. These models can be implemented in the registry databases to help healthcare workers identify patients who may not meet these indicators prospectively and enable timely interventions.

## 1. Introduction

Lung cancer is the fifth most commonly diagnosed cancer in Australia, accounting for 9% of all cancer diagnoses [1], and is the leading cause of mortality, contributing 18% of all cancer deaths [2]. It is the leading cancer burden and contributor of cancer mortality at 8695 per year, which is more than double that of colorectal cancer and three times the prostate cancer mortality [3]. The 5-year survival rate for lung cancer is just 27% [4]. Low survival rates and high treatment costs see lung cancer providing the highest cancer burden in Australia [5].

The timeliness of healthcare delivery represents a critical facet of healthcare excellence. In the context of lung cancer, initiating the first treatment without delay not only holds the potential for curbing disease advancement at a biological level but also brings about patient-centred advantages by alleviating the anxiety and distress linked to treatment postponement [6]. The time from diagnosis to treatment initiation is often seen as an important care management time interval, with reported gaps from diagnosis to treatment initiation ranging from 6 to 45 days, and significant variation observed in how access to care time delays are reported in the literature [7,8].

The effects of timeliness of treatment on survival in NSCLC have revealed mixed results [9,10,11,12,13,14,15]. A systematic analysis of 37 studies indicated a tendency toward decreased survival rates in individuals with advanced-stage disease, while those primarily undergoing surgical treatment showed more favourable outcomes, as reported in reference [16]. In stage 1 patients, a delay in treatment (with intervals exceeding 7 days) led to a decrease in the 5-year survival rate by 9.07% [17]. Conversely, for stage 2 patients who initiated treatment earlier (within an interval of 7 days or less), their 5-year survival rate saw a significant increase of 9.01%. When comparing groups based on the interval between cancer diagnosis and treatment, with the reference group being those treated within 7 days, the adjusted hazard ratio (HR) for mortality increased significantly in the other groups (8–14 days, 15–60 days, and ≥61 days) as the interval time increased (HR 1.04–1.08), with a *p*-value less than 0.05.

The relationship between the timeliness of care among lung cancer patients and mortality has been demonstrated in a previous study [18]. When considering significant individual and area-level risk factors, timely first definitive treatment and multi-disciplinary team meetings (MDM) were found to have an independent association with a reduced likelihood of 2-year all-cause mortality in patients with non-small cell lung cancer (NSCLC) (odds ratio (OR) = 0.73, 95% credible interval (Crl) = 0.56–0.94).

A recent report from Victoria, Australia [19] has identified notable unwarranted clinical variation across hospitals in several lung cancer quality indicators, including the interval between diagnosis and surgical resection, targeting a maximum interval of 14 days. Disparities were observed between metropolitan and regional areas, as well as variations based on the geographical index of relative socio-economic advantage and disadvantage. The report found that while timeliness of care improved (proportion diagnosed within 28 days from referral) from 2019 to 2020, significant variations were observed between metropolitan private and regional sites, as well as in time from diagnosis to resection, which varied between levels of socio-economic disadvantage.

A related previous study examined risk factors associated with the timeliness of care [11]. In the multivariate analysis, factors such as place of birth (Australia versus others), disease stage at diagnosis, notifying hospital type (private versus public), first treatment intent (curative versus non-curative), and palliative care were associated with delay in time from referral to diagnosis. For instance, patients notified in a public hospital experienced a median delay of 31 days compared to 15 days in private hospitals and had a lower mortality hazard ratio of 0.50 (95% CI: 0.41–0.60) compared to those in private hospitals (*p* < 0.001). Regarding the time from diagnosis to initial treatment, independent factors included Eastern Cooperative Oncology Group (ECOG) performance status, disease stage at diagnosis, notifying hospital type (private versus public), and the performance of surgery. For the time from referral to treatment, significant factors included palliative care and treating hospital type (private versus public). The timeliness of lung cancer care is strongly related to the accessibility and availability of healthcare services [20,21,22]. Seeking timely diagnosis and treatment improves survival and treatment outcomes [23,24]. Delay in lung cancer care is strongly linked with increased risk of mortality, distant metastasis, and poor treatment outcomes including sustained anxiety and distress [25,26,27].

Studies are available on predicting the timeliness of lung cancer care using conventional statistical methods such as survival and logistic regression models [11,21,28]. Unlike traditional statistical models, machine learning (ML) algorithms have a superior ability in addressing regression and classifications problems simultaneously to the classical statistical methods [29]. Machine learning techniques have been successfully implemented to analyse large electronic health records, including among patients with type 1 diabetes, to identify risk factors for complications (diabetic ketoacidosis) using decision trees and cross-validation techniques [30], but in that study, the performance of machine learning techniques did not offer significant improvements over the usual logistic regression model when evaluated against the testing dataset. Similarly, machine learning techniques have been used to predict short and long term HbA1c response among patients with type 2 diabetes [31] and who had started insulin treatment with reasonable performance through implementing models such as the elastic net regularisation generalised linear model, support vector machines, and random forests.

In the field of lung cancer, machine learning techniques have been used to predict early lung cancer using metabolic biomarkers and clinical information with good performance (AUC = 0.81) [32], derive a clinical prediction model for pulmonary metastasis [33], predict radiation-induced toxicities among lung cancer patients undergoing radiotherapy [34], estimate lung disease mortality from chest x-rays and other clinical and demographic information [35], and estimate lung cancer survival time intervals [36]. In the current era of personalised precision medicine in lung cancer, machine learning has shown encouraging capability in the integration of disparate data elements from complex datasets, including clinical characteristics and patient demographics, diagnostic medical imaging and molecular and genomic status to enhance histopathological characterisation, prognostic accuracy, prediction of metastasis, and clinical decision making [37,38,39].

To date, there is scant information on the capability of machine learning techniques in the evaluation and prediction of the quality of care in lung cancer. This project is the first to employ such novel techniques in this field.

## 2. Materials and Methods

### 2.1. Setting

This study obtained deidentified lung cancer data from the Victorian Lung Cancer Registry (VLCR) between 2011 and 2022 [19]. The registry collects data from 19 Victorian health services covering 40 hospitals, encompassing around 85% of Victorian lung cancer notifications. The aim of VLCR is to collect real-world observational data from patients and provide risk-adjusted benchmarked quality indicator reports to healthcare practitioners with a view to improving healthcare delivery and improving the health and well-being of lung cancer patients. Specifically, the VLCR report aims to provide institutional benchmarking to identify multiple opportunities for quality improvements in lung cancer detection, management, and treatment at hospitals. Patients were included if they were at least 18 years old and presented with incident non-small cell lung cancer (NSCLC) and small cell lung cancer (SCLC) based on either a clinical or pathological diagnosis. Exclusion criteria included the presence of secondary lung cancer, thymoma, or mesothelioma. Ethics approval for this study was obtained from Monash University Human Research Ethics Committee (Project number: 39010).

### 2.2. Outcome Measures

1. The interval between initial referral for management and diagnosis (“referral to diagnosis”): Target ≤ 28 days (QI 1).
2. The interval between diagnosis and initial surgery, chemotherapy, radiotherapy, or referral to palliative care (“diagnosis to initial definitive management”): Target ≤ 14 days (QI 2).
3. Time from diagnosis date to surgical resection date among patients with NSCLC: Target ≤ 14 days (QI 3).
4. The interval between referral and initial definitive management: Target ≤ 42 days (QI 4).

The cut-offs for these timelines are based on official Australian guidelines [40]. The referral date is the date recorded on the referral letter to the hospital or diagnosing clinician. The date of diagnosis is the date a pathological test confirms a primary lung cancer. If a patient does not have a pathological test to confirm a primary lung cancer, the date of diagnosis is the date a clinical test confirmed a primary lung cancer, supported by medical correspondence confirming a primary lung cancer. See Appendix A for definitions.

### 2.3. Predictive Features

This analysis encompassed various patient phenotypic predictive risk features, including sex (male versus female), age at diagnosis, country of birth (Australia versus others), preferred language (English versus others), smoking status, tumour, node, metastasis (TNM), clinical stage of primary tumour at diagnosis based on the International Association for the Study of Lung Cancer classification [41], Eastern Cooperative Oncology Group (ECOG) performance status (0: good to 4: poor) [42], lung cancer type (small cell lung cancer versus NSCLC), notifying hospital, diagnosing hospital, and type of hospital (private versus public). These data are collected from participating hospitals’ medical records, following a 2-week opt-out period for patients.

At the patient level, the only available residential information was the postal areas (POA) derived from the VLCR. POAs are smaller spatial units compared to statistical areas (SA2) and can be linked to census population data directly from the Australia Bureau of Statistics (ABS). POA has been identified as an appropriate scale for evidence-based tools for effectively targeting policy interventions. From the ABS 2016 census [43,44], data for the analysis including the index of relative socio-economic disadvantage (IRSD) and the remoteness index were obtained. The IRSD serves as a surrogate indicator of area-level socio-economic status (SES). A low IRSD score in a given POA indicates a higher proportion of relatively disadvantaged individuals. The IRSD was categorized into quartiles, while the remoteness index was categorized into metropolitan (major cities) and regional areas (inner regional, outer regional, and remote/very remote Australia). Other measures of SES such as IRSAD, IER, and IEO were also included in the prediction model. To improve predictive capabilities, continuous variables were standardised by subtracting the variables from their means and dividing them by their standard deviations. All the predictive features listed above were included in the model, as they were deemed, a priori, to be significant predictors of timeliness of care.

### 2.4. Statistical Methods

Supervised machine learning techniques were implemented to classify patients into the 4 quality indicators listed above using the machine learning techniques described below. The models were set up and run through a user-written command in Stata “c_ml_stata_cv”, which implements the Python Scikit-learn tools via a Stata/Python integration function [45]. The following machine learning models were studied: decision tree, boosting, random forests, regularized multinomial, neural network, naive Bayes, nearest neighbour, and support vector machine, and the hyper-parameters for each machine learning technique were optimized via greed search using 10-fold cross-validation techniques (described in detail below).

### 2.5. Machine Learning Methods

1. Decision Trees [46,47]

Decision trees are supervised machine learning tools that split data based on parameters into nodes and leaves to construct a tree-like model. Information gain is used for node division. Key parameters include the following: Maximum depth: controls tree depth for capturing complex relationships while preventing overfitting. Minimum samples to split: requires a certain sample count before node splitting, preventing premature splits. Minimum samples in a leaf: sets the minimum samples needed in a leaf node, curbing overfitting. Maximum leaf nodes: sets the upper limit for leaf nodes, aiding in avoiding overfitting. Splitting criterion: chooses metrics like “GINI” or “entropy” to assess split quality based on impurity or uncertainty. Class weight: adjusts class influence to manage imbalanced data by assigning weights, with “balanced” being an automated option.

2. Boosting [46,47]

Boosting is an ensemble machine learning method that sequentially fits trees to residuals from the current model, combining them into a strong learning model. In gradient boosting, only variables enhancing prediction accuracy beyond a threshold are used. Key parameters include the following: Number of estimators: Combines weak learning models. More trees can boost performance but risk overfitting. Learning rate: lower rates prevent overfitting, but more estimators might be needed. Maximum depth: Limits boosting tree depth. Higher depth can overfit. Usually, there’s a trade-off between learning rates and number of trees.

3. Random Forests [46,47]

Random forests employs multiple decision trees and averages their predictions. Cross validation selects variables and cut-offs. Each tree considers a random subset of predictors for decorrelation. Tunable parameters for model optimization include the following: number of trees, maximum, minimum samples to split, splitting criterion.

4. Regularized Multinomial

Regularized multinomial models like regularized logistic regression or regularized multinomial naive Bayes can be optimized by adjusting key parameters: Regularization strength: Controls regularization level. Smaller C enhances regularization, preventing overfitting. Larger C weakens regularization for better training data fit, but risks overfitting. Penalty type. L1 ratio: Chooses “l1” or “l2” regularization. L1 adds absolute coefficient penalty, encouraging sparse models. L2 adds squared coefficient penalty, yielding smoother models.

5. Neural Network [48,49]

Neural networks consist of hidden layers linking predictors to outcomes, each with nodes passing signals using activation functions. Learning adjusts weights to minimize loss. Key parameters for optimization: Number of hidden layers: more layers capture complexity but risk overfitting. Number of neurons per layer: more neurons capture complexity but risk overfitting. Activation function: (e.g., sigmoid, ReLU, tanh) affects neuron output, influencing pattern capture. Learning rate: larger rates speed convergence but risk instability. Dropout rate: Probability of neuron dropout in training. Reduces overfitting by limiting individual neuron impact. Regularization strength (alpha): Controls regularization extent. Prevents overfitting by penalizing large weights.

6. Naive Bayes

Naive Bayes models can be optimized by adjusting key parameters: Smoothing parameter: Manages probability smoothing. Lower alpha means less smoothing, risking overfitting. Higher alpha increases smoothing, aiding prevention of overfitting and rare feature impact. Prior probabilities: Sets initial class probabilities. Defaults based on training data frequencies. Adjust for prior knowledge of class distribution.

7. K-Nearest Neighbour

The main parameter for the k-nearest neighbour (k-NN) algorithm: k: The count of nearest neighbours considered for prediction. Its choice impacts the bias–variance trade-off. Smaller k leads to flexible, low-bias but high-variance models. Larger k yields rigid, high-bias but low-variance models. k’s value is tuned through cross validation or other methods.

8. Support Vector Machine [47]

Support vector machine (SVM) models create hyperplanes to separate observations, linearly or non-linearly via kernels. The parameters to optimize an SVM’s performance include the following: Kernel type (kernel): Chooses a function to transform data for hyperplane separation. Options are linear, polynomial, and radial basis function (RBF). Choice depends on data nature and problem. Regularization parameter: balances margin maximization and classification error minimization. Smaller C: wider margin, more misclassifications; larger C: narrower margin, fewer misclassifications. Kernel-specific parameters: Gamm: controls RBF kernel’s Gaussian width. Smaller gamma: wider, smoother decision boundaries; larger gamma: narrower, complex boundaries.

### 2.6. Model Evaluation

Prediction is improved via a 10-fold cross validation (re-sampling), which is used to optimally tune the various parameters of the individual machine learning methods, through minimising the test (or out-of-sample) classification errors. Each of the machine learning models requires at least 1 tuning parameter to be specified. The aim is to determine a model which is parsimonious and least complex. Machine learning models were assessed based on their accuracy. Missing data based on the outcome variable were excluded. For the predictive features, we created a category for missing data and included the category in the model to preserve sample size.

### 2.7. Parameter Tuning

**Figure 1:**
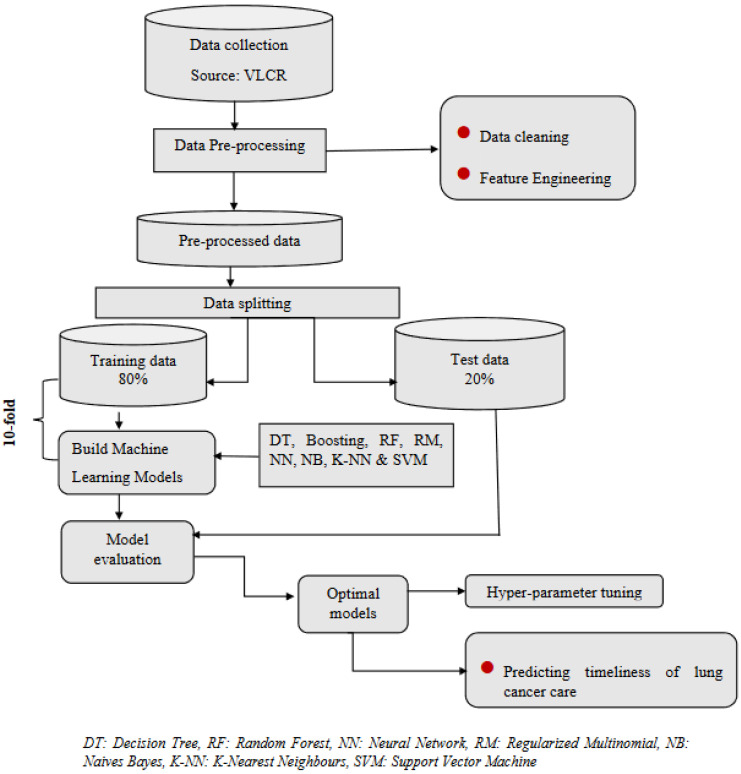
Conceptual framework of data preparation, splitting, and analysis applied.

Data were randomly split into 2 sets: 80% for a training dataset, where the model was tuned and developed, and then the final model was tested on a 20% dataset. Throughout the training process, each model underwent 10-fold cross validation. This involved splitting the training set into a training subset and a validation subset with a ratio of 10:1 to fine-tune the hyperparameters by minimising the out-of-sample classification errors. The parameters were selected from a list of values through grid search from exploratory analysis, and the values for each machine learning method and parameters are shown in Appendix B. Following the optimization of tuning parameters, the model underwent evaluation using a tenfold cross-validation approach applied repeatedly ten times. This rigorous process involved dividing the data into ten subsets or folds, with the model being trained and assessed iteratively, rotating through each fold as a test set while the remaining nine folds were utilised for training. The ultimate accuracy metric was established by amalgamating the outcomes from the ten cross-validated models through the use of the Area Under the Receiver Operating Characteristic Curve (AUC-ROC). This curve graphically illustrates the relationship between the true positive rate and the false positive rate and ranges between zero and one, with a higher value signifying better predictive performance. The decision to prioritize accuracy as the optimizing metric was driven by the desire to ascertain the optimal threshold value for prediction determination, where the AUC provides an overall snapshot of classification performance for our study. The clinical significance of these AUCs were interpreted using these cut-offs (<0.20 = poor, 0.21–0.40 = fair, 0.41–0.60 = moderate, 0.61–0.80 = good, ≥0.80 = very good) [50]. A schematic conceptual model of the design, data wrangling, analysis and reporting is provided in Figure 1.

## 3. Results

**Figure 2:**
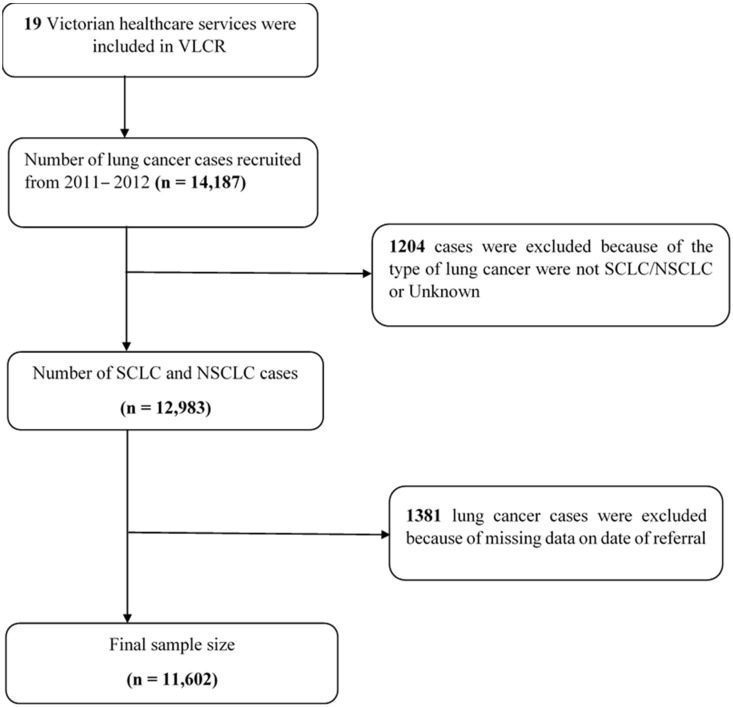
Flowchart of patient inclusion/exclusion criteria and final cohort for study.

Out of the initial 14,720 lung cancer patients registered with the VLCR, after excluding those patients diagnosed interstate and missing lung cancer type and date of referral, a total of 11,602 patients were included in our study (Figure 2).

The demographic and clinical characteristics of the cohort in its entirety (11,602) and stratified by adherence to QI 1 are shown in Table 1 below. Slightly more than half (56%) were male with a mean age of 69 years (SD = 11). In terms of ECOF status at diagnosis, most were able, with category 1 (24%) and category 2 (30%). The vast majority spoke English as a first language (90%). In terms of smoking status, 51% were ex-smokers and 35% current smokers. In terms of lung cancer type, most (88%) were of the more serious NSCLC type, with 46% presenting at the stage 4 clinical stage at diagnosis.

In terms of adherence to QI 1 (i.e., referral to diagnosis within 28 days), 8008 patients (69%) met the criteria. There were significant differences in demographics and clinical and contextual factors between those who met/did not meet QI 1 (Table 1). A higher proportion of patients who met QI 1 were males, were younger, had lower ECOG scores, were smokers, lived in outer regional areas, had a higher stage, 4, of disease, and were Australian born. SES indices such as IRSD, IEO, and IRSAD were also significantly higher among those who met QI 1. For instance, 62% of those who met QI 1 were Australian born compared to 58% of those born elsewhere, and this difference was statistically significant (*p* < 0.001, chi-squared test).

The figures in Appendix C highlight the optimal tuning of parameters for the eight different machine learning methods utilised in the study. The optimal combination of parameters for each machine learning model and their performance for both the training and testing datasets are shown in Table 2. The support vector machine learning method (margin parameter C = 1, gamma = 1) performed the best among all machine learning models, in terms of a testing AUC of 0.89 and training AUC of 0.99. The classification error rates (CER) for training and validation datasets were 0.2% and 0.1%, respectively. The nearest neighbour model (# of neighbours = 100, kernel = distance) performed well as well, with a testing AUC = 0.85 and testing CER = 0.1%. The boosting model performed third best (tree depth 15, number of trees = 150, and learning rate = 0.3), with an AUC = 0.83. These machine learning models performed much better than the traditional logistic regression model (training and testing AUC = 0.73).

Appendix D shows the relationship between the various demographic, clinical, and socio-economic characteristics and meeting the individual quality indicators 2, 3, and 4. For QI 2, significant associations were found across all variables, except for smoking status and Australian born. A significantly higher proportion of those who met QI 2 had SCLC (23%) compared to NSCLC (6%), *p* < 0.001. For QI 3, ECOG status at diagnosis, smoking status, remoteness location of residence, clinical stage, and socio-economic location were all significant predictors. A higher proportion of patients who met QI 3 resided in major cities (72%) compared to those who did not meet the quality indicator (64%), *p* < 0.001. Finally, for QI 4, all features were significant, except for sex, smoking status, and Australian born. Those who met QI 4 stayed in a higher IRSD location (1004) compared to those who did not meet this indicator (982), *p* < 0.001).

**Figure 3:**
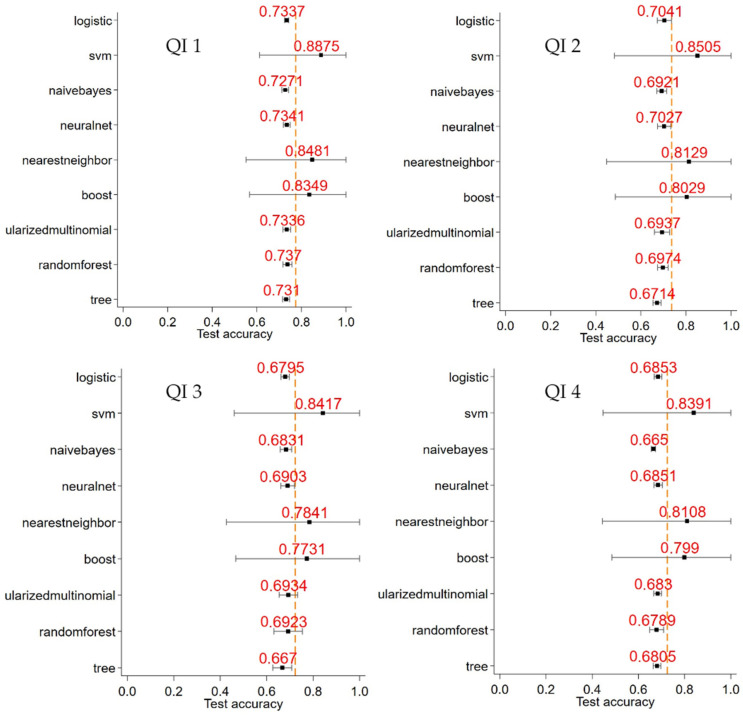
Out-of-sample area under the curve comparisons of machine learning methods for quality indicators 1 to 4.

This study shows that in terms of meeting the targeted timeliness quality indicators, for QI 1: overall proportion of “time from referral date to diagnosis being ≤ 28 days”, QI 2: proportion where “time from diagnosis date to first treatment date (any intent) being ≤ 14 days”, QI 3: proportion where “time from diagnosis date to surgical resection date being ≤ 14 days”, and QI 4: proportion where “time from referral date to first treatment (any intent) being ≤ 42 days”, the overall proportions across the whole cohort were 69%, 41%, 60%, and 49%, respectively. Figure 3 summarises the out-of-sample AUCs for the various machine learning models for quality indicators 1 to 4. The support vector machine, nearest neighbour, and boosting trees performed consistently well, compared to the logistic regression model, for these quality indicators.

## 4. Discussion

This study has found that machine learning techniques can provide better classification of lung cancer patients in terms of meeting key quality indicators for timeliness of care. Specifically, machine learning models such as support vector machines, k-nearest neighbour, and boosting trees fared much better than the logistic regression model. In terms of clinical significance, the performance of these models can be rated as having very good discriminatory properties (AUC > 0.80) compared to the traditional logistic regression model (rated good). The AUC of 0.89 was also higher compared to another study involving a large dataset of electronic health record analysis, which found AUCs ranging from 0.64 to 0.73 [51], when trying to identify patients with delays in starting cancer treatment using patient demographic, clinical, and neighbourhood socio-economic indices. Timeliness of care is important, as it has been shown to be associated with survival, and interventions to improve on the timeliness and completeness of cancer investigations and treatment such as a customised “OnkoNetwork” patient navigation program have been demonstrated to provide for a large survival benefit (HR = 0.63, *p* = 0.039) [52], but the first important step is to better classify which groups of patients would benefit from such interventions.

The incidence of timely diagnosis of lung cancer was 69%. This is lower than the study reported in Jordan [53]. This could be due to the difference in cut-off point used to define the timely diagnosis of lung cancer, and the period of study. The majority of the patients had delayed treatment after diagnosis and referral. The possible reason might be due to diagnostic investigations and treatment being very expensive [54,55,56], which meant they couldn’t be afforded by the majority of the patients. The timeliness of lung cancer care is influenced by patient-related, physician-related, and system-related factors [57]. In the majority of lung cancer cases, during the initial phases, the lung cancer patients commonly have non-specific symptoms, and this could have made them delay in seeking healthcare. Another explanation could be due to the impact of the coronavirus (COVID-19) pandemic, as during the pandemic, medical services were shifted to COVID-19 patients [58]. This could contribute to the delay in lung cancer presentation, diagnosis, and treatment [59,60].

Demographic, clinical, and area-level determinants such as SEIFA and remoteness have a significant impact on the timeliness of lung cancer care. Age and IRSAD were found to be important predictors of timeliness of lung cancer care. These findings are in line with previous studies [57,61,62,63,64], and given that the costs of diagnosis and treatment are rising unexpectedly [65,66], lung cancer patients with the lowest socio-economic status may not be able to afford the high costs of cancer diagnosis and treatment [67,68]. Lung cancer patients with better incomes have a higher chance of seeking early diagnosis and treatment services [69,70]. Younger age groups usually perceive that they are capable of coping with medical conditions and identify themselves as not being susceptible; this could have made them visit hospitals at a later stage than older age groups did. Additionally, usually, older people have comorbidities and manifest more clinical symptoms of lung cancer than young people do [71].

This study has the following strengths. Firstly, before testing and training the machine learning algorithms, appropriate pre-processing of the dataset was performed to avoid errors. Secondly, the predictors used in the model were biologically plausible and screened by an expert oncologist. Moreover, to the best of our knowledge, this is the first study applying the novel machine learning algorithms for the prediction of the timeliness of lung cancer care. The analysis has some limitations. Even though eight different machine learning methods were utilised, it is acknowledged that these are not comprehensive and that other techniques are available (e.g., deep learning, lasso, etc.). The aim was to provide researchers with familiarity with the Stata (Version 17.0, Stata Corp, College Station, TX, USA) software’s access to such machine learning tools and, hence, we were unable to compare against the other techniques. Garavand and colleagues [47] provide a brief comparison among the various machine learning methods: neural networks are one of the widely used machine learning algorithms; survey vector machines, developed by Vladimir Vapnik, have been successfully applied to many classification and forecasting studies; random forests, introduced in 2001, is a highly recommended classifier when dealing with overfitting and underfitting; KNN is one of the simplest and is preferred over other classifiers due to its simplicity and high convergence speed. In this study, neural networks performed similarly to random forests, and one possible reason could be due to the fact that neural networks work better with larger datasets (i.e., >100,000 observations) [72]. A formal comparison of the methods would be beyond the scope of this research.

For a similar reason, this study was only able to optimize selected parameters within each machine learning model, based on their availability in the user-written Stata code. We are planning to expand on the Stata code to enable fine-tuning other parameters in future work. The 95% confidence intervals for the AUCs for some of the models were also relatively wide. This could potentially be due to a smaller sample size for the testing dataset and selecting from a narrow grid search for the parameters for these models. In a future work, the list of machine learning methods can be expanded and the grid search widened for the parameters. It is also noted that there is an imbalance in numbers for quality indicator 1, namely 8008 patients with a referral to diagnosis within 28 days and 3594 patients with an interval > 28 days, and this may affect the performance of some of the machine learning techniques utilised in this study. This is acknowledged as an area for further work, but a recent study [72], which over-sampled the target class (or outcome) to address the potential limitation, showed that taking this approach did not improve the performance of their compared models.

In terms of the dataset, a real-world multicentre registry dataset from Victoria, Australia was analysed. While the data are comprehensive across regions, missing data are inherent in large observational studies such as ours. Missing data on outcome (e.g., referral date) were excluded in the analysis, as the models utilised could not accommodate missing data, but the impact on the results should be minimal due to the relatively small amounts of missing data (e.g., referral date missing for n = 1381/12,983). For predictive features, a category for missing data was created, so that was not an issue. Future work will evaluate the most appropriate way to deal with missing data (e.g., imputation). The VLCR is also not 100% in terms of population coverage, but completeness and accuracy of recruitment of the eligible population has been assessed on a scheduled basis by comparing data from the clinical registry with other data sources such as the Victorian Cancer Registry, the Victorian Admitted Episode Data, and hospital clinical record data [19]. Most of the predictive features utilised in this study such as the clinical variables may not be subject to bias, but some such as smoking status could be poorly reported and cause misclassification, and this is acknowledged as a limitation.

The provision of timely care in lung cancer management is a critical measure of quality of care [73]. Delay in care provides the biological risk of disease progression and the loss of opportunity of provision of curative-intent treatment as the cancer stage progresses. The timing of a new lung cancer diagnosis is also a period of high-level anxiety, distress, and social disruption likely to be beneficially impacted by timely care and the absence of delay.

## 5. Future Directions

This study’s results have important clinical implications. It may be possible to develop an app or integrate these models into hospital clinical information systems, so that when a few key pieces of patient demographic and clinical information are keyed into the database, the models can help to “predict” patients who may experience a delay in referral to diagnosis or treatment, thus prompting case management, triage, or escalation of due follow-up. The models can also be fine-tuned or validated to work with vulnerable populations. Future research could also examine other tools such as deep learning, and also optimize more machine learning methods’ parameters and address the issue of missing data. The structure of the design, analysis, and reporting of the data in this research could lead to different results when configurations and tools other than those described above are used. In terms of practical implementation of such tools in clinical practice, a cost-effectiveness analysis would be beneficial in terms of whether the additional costs of integrating such models in hospitals’ clinical data collection and management systems would be outweighed in terms of improvements in patients’ health outcomes through the provision of timely lung cancer care, and this would be an interesting follow-up work.

## 6. Conclusions

This research has demonstrated that machine learning models can be successfully implemented to classify timeliness of care among lung cancer patients. The findings have implications in terms of patient care, as clinicians can utilise such tools to evaluate which patients should be followed up closely or case managed to pre-empt delay in diagnosis and treatment to ultimately improve their clinical outcomes. Plans are set in place for a test model to be showcased to sites via the steering committee of the VLCR.

## Figures and Tables

**Table 1:** Demographic and clinical characteristics of lung cancer cohort, and split by adherence to quality indicator 1 (referral to diagnosis within 28 days).

Variables	QI 1 No	QI 1 Yes	Whole Cohort	*p*-Value	Test
N	3594	8008	11,602		
Sex				0.027	Pearson’s chi-squared
Male	1969 (54.8%)	4564 (57.0%)	6533 (56.3%)		
Female	1625 (45.2%)	3444 (43.0%)	5069 (43.7%)		
Age, mean (SD)	70.1 (10.0)	68.9 (10.9)	69.3 (10.6)	<0.001	Two-sample *t* test
ECOG status at diagnosis				<0.001	Pearson’s chi-squared
0—Fully active, able to carry on all normal activity without restriction	1005 (28.0%)	1809 (22.6%)	2814 (24.3%)		
1—Restricted in physically strenuous activity but ambulatory and able to carry out light work	955 (26.6%)	2467 (30.8%)	3422 (29.5%)		
2—Ambulatory and capable of all self-care but unable to carry out any work activities	282 (7.8%)	850 (10.6%)	1132 (9.8%)		
3—Capable of only limited self-care, confined to bed or chair more than 50% of waking hours	105 (2.9%)	393 (4.9%)	498 (4.3%)		
4—Completely disabled. Not able to care for self. Totally confined to bed or chair	13 (0.4%)	55 (0.7%)	68 (0.6%)		
8—Not available at time of presentation	4 (0.1%)	17 (0.2%)	21 (0.2%)		
9—Not stated	1230 (34.2%)	2417 (30.2%)	3647 (31.4%)		
English as first language				<0.001	Pearson’s chi-squared
No	414 (11.5%)	712 (8.9%)	1126 (9.7%)		
Yes	3180 (88.5%)	7296 (91.1%)	10,476 (90.3%)		
Smoking status				0.001	Pearson’s chi-squared
Current smoker	1212 (33.7%)	2892 (36.1%)	4104 (35.4%)		
Ex-smoker	1863 (51.8%)	4005 (50.0%)	5868 (50.6%)		
Never smoker	455 (12.7%)	908 (11.3%)	1363 (11.7%)		
Not stated	64 (1.8%)	203 (2.5%)	267 (2.3%)		
Remoteness				0.010	Pearson’s chi-squared
Inner regional Australia	954 (26.5%)	2166 (27.1%)	3120 (26.9%)		
Major cities of Australia	2410 (67.1%)	5210 (65.1%)	7620 (65.7%)		
Outer regional Australia	224 (6.2%)	624 (7.8%)	848 (7.3%)		
Remote Australia	6 (0.2%)	7 (0.1%)	13 (0.1%)		
Clinical stage				<0.001	Pearson’s chi-squared
Stage 1	1005 (28.0%)	612 (7.6%)	1617 (13.9%)		
Stage 2	423 (11.8%)	468 (5.8%)	891 (7.7%)		
Stage 3	509 (14.2%)	1333 (16.6%)	1842 (15.9%)		
Stage 4	845 (23.5%)	4474 (55.9%)	5319 (45.8%)		
Cannot be assessed	812 (22.6%)	1121 (14.0%)	1933 (16.7%)		
Lung cancer type				<0.001	Pearson’s chi-squared
NSCLC	3344 (93.1%)	6836 (85.4%)	10,180 (87.8%)		
SCLC	249 (6.9%)	1172 (14.6%)	1421 (12.2%)		
Australian born				<0.001	Pearson’s chi-squared
Other/Not stated	1497 (41.7%)	3027 (37.8%)	4524 (39.0%)		
Australia	2097 (58.3%)	4981 (62.2%)	7078 (61.0%)		
Index of relative socio-economic disadvantage, mean (SD)	997.4 (70.7)	1001.9 (70.0)	1000.5 (70.2)	0.001	Two-sample *t* test
Index of economic resources, mean (SD)	990.1 (60.7)	992.4 (60.2)	991.7 (60.3)	0.054	Two-sample *t* test
Index of education and occupation, mean (SD)	1003.6 (85.2)	1008.9 (86.7)	1007.2 (86.3)	0.002	Two-sample *t* test
Index of relative socio-economic advantage and disadvantage, mean (SD)	995.9 (75.2)	1000.6 (76.1)	999.1 (75.9)	0.002	Two-sample *t* test

**Table 2:** Optimal parameters for selected machine learning models based on 10-fold cross validation for quality indicator 1.

Model	Parameters	Training CER	Validation CER	Training AUC	Testing AUC
Decision trees	Tree depth = 5	25.90%	28.20%	0.74	0.71
Random forests	Tree depth = 10	21.60%	23.40%	0.79	0.74
	# splitting features = 10				
	# of trees = 100				
Regularized multinomial	Penalisation parameter, alpha = 0.01	26.60%	27.30%	0.73	0.73
	Elastic parameter (regularization = 0)				
Boosting	Tree depth = 15	0.10%	0.10%	0.99	0.83
	# of trees = 150				
	Learning rate = 0.3				
Nearest neighbour	# of neighbours = 100	0.10%	0.10%	0.99	0.85
	Kernel = distance				
Neural networks	# of layers = 4	31.00%	31.10%	0.73	0.73
	# of neurons = 1				
	L2 penalisation = 0.5				
Naïve Bayes	Variance smoothing = 0.001	35.20%	34.80%	0.73	0.73
Support vector machine	Margin parameter, C = 1	0.20%	0.10%	0.99	0.89
	Inverse distance, gamma = 1				
Logistic regression	NA	26.50%	26.60%	0.73	0.73

Note: CER: classification error rate. AUC: Area under the curve. NA denotes Not Applicable.

## Data Availability

Restrictions apply to the availability of the data. Data was obtained from the Victorian Lung Cancer Registry and is available to researchers once they make a formal application to the registry and it is approved. https://vlcr.org.au/.

## Appendix A. Definition of Quality Indicators

**Indicator**	**Operational Definition**
QI 1	Number of patients where time from referral date to diagnosis is ≤28 days (numerator). Number of patients in Registry with a referral date available (denominator).
QI 2	Number of patients where time from diagnosis date to first treatment date (any intent) is ≤14 days (numerator). Number of patients in Registry receiving any intent anti-cancer treatment with a defined date (denominator).
QI 3	Number of patients with NSCLC where time from diagnosis date to surgical resection date is ≤14 days (numerator). Number of NSCLC patients in Registry undergoing surgical resection with defined dates available (denominator).
QI 4	Number of patients where time from referral date to first treatment (any intent) is ≤42 days (numerator). Number of patients in Registry undergoing anti-cancer treatment with referral and treatment date available (denominator).

## Appendix B. Machine Learning Models and the Parameters Specified in the Grid Search

**Machine Learning Method**	**Parameters**	**Grid Search**						
Decision trees	Tree depth	5	6	7	8	9	10	100
Random forests	Tree depth	10	11	12	13	14	15	
	# of splitting features	5	10	20				
	# of trees	10	100	1000				
Regularised multinomial	L1 ratio	0	0.5	1				
	Alpha	0.01	0.05	0.5				
Boosting	Tree depth	5	10	15	20			
	# of trees	100	150	200				
	Learning rate	0.01	0.1	0.2	0.3			
Nearest neighbour	# of neighbours	10	100	150	200			
	Kernel	Uniform	Distance					
Neural network	# of neurons L1	2	4	6	8			
	# of neurons L2	1	2	3	4			
	Alpha	0.5	1	1.1	1.5			
Support vector machines	Margin parameter, C	0.01	0.1	0.5	1	10		
	Gamma	0	1					

## Appendix C. Optimal Tuning of Machine Learning Method Parameters Based on Cross-Validation Classification for Quality Indicator One (QI 1)

(a) **Decision trees**

(b) **Random forests**

(c) **Regularised multinomial**

(d) **Boosting**

(e) **Nearest neighbour**

(f) **Neural networks**

(g) **Naïve Bayes**

(h) **Support vector machines**

## Appendix D. Descriptive and Demographic Characteristics Stratified by Quality Indicators 2 to 4

**Variables**	**QI 2 No**	**QI 2 Yes**	***p*-Value**	**QI 3 No**	**QI 3 Yes**	***p*-Value**	**QI 4 No**	**QI 4 Yes**	***p*-Value**
N	5785	4063		1098	1614		5045	4803	
Sex			0.012			0.14			0.25
Male	3286 (56.8%)	2204 (54.2%)		582 (53.0%)	809 (50.1%)		2841 (56.3%)	2649 (55.2%)	
Female	2499 (43.2%)	1859 (45.8%)		516 (47.0%)	805 (49.9%)		2204 (43.7%)	2154 (44.8%)	
Age, mean (SD)	69.1 (10.5)	67.6 (10.2)	<0.001	68.0 (9.7)	68.2 (9.4)	0.62	69.6 (10.1)	67.3 (10.6)	<0.001
ECOG status at diagnosis			<0.001			<0.001			<0.001
0	1617 (28.0%)	1057 (26.0%)		474 (43.2%)	541 (33.5%)		1474 (29.2%)	1200 (25.0%)	
1	1997 (34.5%)	1056 (26.0%)		244 (22.2%)	257 (15.9%)		1563 (31.0%)	1490 (31.0%)	
2	572 (9.9%)	322 (7.9%)		21 (1.9%)	33 (2.0%)		439 (8.7%)	455 (9.5%)	
3	166 (2.9%)	135 (3.3%)		5 (0.5%)	11 (0.7%)		117 (2.3%)	184 (3.8%)	
4	10 (0.2%)	14 (0.3%)		0 (0.0%)	1 (0.1%)		6 (0.1%)	18 (0.4%)	
Not available	9 (0.2%)	9 (0.2%)		1 (0.1%)	3 (0.2%)		8 (0.2%)	10 (0.2%)	
Not Stated	1414 (24.4%)	1470 (36.2%)		353 (32.1%)	768 (47.6%)		1438 (28.5%)	1446 (30.1%)	
English as first language			0.002			0.34			<0.001
No	603 (10.4%)	347 (8.5%)		107 (9.7%)	140 (8.7%)		538 (10.7%)	412 (8.6%)	
Yes	5182 (89.6%)	3716 (91.5%)		991 (90.3%)	1474 (91.3%)		4507 (89.3%)	4391 (91.4%)	
Smoking status			0.090			0.003			0.26
Current smoker	2015 (34.8%)	1452 (35.7%)		360 (32.8%)	441 (27.3%)		1749 (34.7%)	1718 (35.8%)	
Ex-smoker	2982 (51.5%)	2000 (49.2%)		559 (50.9%)	843 (52.2%)		2599 (51.5%)	2383 (49.6%)	
Never smoker	687 (11.9%)	531 (13.1%)		164 (14.9%)	292 (18.1%)		610 (12.1%)	608 (12.7%)	
Not stated	101 (1.7%)	80 (2.0%)		15 (1.4%)	38 (2.4%)		87 (1.7%)	94 (2.0%)	
Remoteness			<0.001			<0.001			0.008
Inner regional Australia	1660 (28.7%)	1004 (24.7%)		317 (28.9%)	355 (22.0%)		1436 (28.5%)	1228 (25.6%)	
Major cities of Australia	3676 (63.5%)	2774 (68.3%)		697 (63.5%)	1156 (71.6%)		3225 (63.9%)	3225 (67.2%)	
Outer regional Australia	443 (7.7%)	279 (6.9%)		82 (7.5%)	101 (6.3%)		378 (7.5%)	344 (7.2%)	
Remote Australia	6 (0.1%)	5 (0.1%)		2 (0.2%)	2 (0.1%)		6 (0.1%)	5 (0.1%)	
Clinical stage			<0.001			<0.001			<0.001
Stage 1	836 (14.5%)	704 (17.3%)		478 (43.5%)	677 (41.9%)		1154 (22.9%)	386 (8.0%)	
Stage 2	546 (9.4%)	279 (6.9%)		249 (22.7%)	221 (13.7%)		596 (11.8%)	229 (4.8%)	
Stage 3	1300 (22.5%)	370 (9.1%)		156 (14.2%)	118 (7.3%)		1009 (20.0%)	661 (13.8%)	
Stage 4	2437 (42.1%)	1828 (45.0%)		23 (2.1%)	20 (1.2%)		1444 (28.6%)	2821 (58.7%)	
Cannot be assessed	666 (11.5%)	882 (21.7%)		192 (17.5%)	578 (35.8%)		842 (16.7%)	706 (14.7%)	
Lung cancer type			<0.001						<0.001
NSCLC	5465 (94.5%)	3146 (77.4%)					4776 (94.7%)	3835 (79.8%)	
SCLC	320 (5.5%)	917 (22.6%)					269 (5.3%)	968 (20.2%)	
Australian born			0.95			0.18			0.17
Other/Not stated	2242 (38.8%)	1572 (38.7%)		431 (39.3%)	675 (41.8%)		1987 (39.4%)	1827 (38.0%)	
Australia	3543 (61.2%)	2491 (61.3%)		667 (60.7%)	939 (58.2%)		3058 (60.6%)	2976 (62.0%)	
Index of relative socio-economic disadvantage, mean (SD)	998.3 (70.7)	1005.2 (68.3)	<0.001	998.4 (72.5)	1009.8 (67.4)	<0.001	998.2 (69.6)	1.004.3 (69.9)	<0.001
Index of economic resources, mean (SD)	990.3 (60.2)	995.4 (60.2)	<0.001	991.1 (58.9)	998.9 (58.2)	<0.001	990.7 (59.5)	994.3 (60.9)	0.003
Index of education and occupation, mean (SD)	1004.1 (85.9)	1012.0 (85.5)	<0.001	1004.7 (87.6)	1017.1 (85.8)	<0.001	1003.1 (84.3)	1011.9 (87.2)	<0.001
Index of relative socio-economic advantage and disadvantage, mean (SD)	996.1 (76.0)	1004.3 (74.6)	<0.001	996.5 (77.9)	1009.2 (74.9)	<0.001	995.5 (74.5)	1003.6 (76.3)	<0.001

## References

[1] Australian Government Cancer Australia (2022). Lung Cancer in Australia Statistics.

[2] Cancer Council Victoria (2023). Lung Cancer Statistics and Trends.

[3] Australian Institute of Health and Welfare (2021). Australian Cancer Incidence and Mortality (ACIM) Books.

[4] Cancer Council Victoria (2021). Victorian Cancer Registry. Cancer in Victoria.

[5] Goldsbury D.E., Weber M.F., Yap S., Rankin N.M., Ngo P., Veerman L., Banks E., Canfell K., O’Connell D.L. (2020). Health services costs for lung cancer care in Australia: Estimates from the 45 and up Study. PLoS ONE.

[6] Risberg T., Sorbye S.W., Norum J., Wist E.A. (1996). Diagnostic delay causes more psychological distress in female than in male cancer patients. Anticancer Res..

[7] Guirado M., Fernandez Martin E., Fernandez Villar A., Navarro Martin A., Sanchez-Hernandez A. (2022). Clinical impact of delays in the management of lung cancer patients in the last decade: Systematic review. Clin. Transl. Oncol..

[8] Jacobsen M.M., Silverstein S.C., Quinn M., Waterston L.B., Thomas C.A., Benneyan J.C., Han P.K. (2017). Timeliness of access to lung cancer diagnosis and treatment: A scoping literature review. Lung Cancer.

[9] Cushman T.R., Jones B., Akhavan D., Rusthoven C.G., Verma V., Salgia R., Sedrak M., Massarelli E., Welsh J.W., Amini A. (2021). The Effects of Time to Treatment Initiation for Patients With Non-small-cell Lung Cancer in the United States. Clin. Lung Cancer.

[10] Di Girolamo C.W.S., Gildea C., Benitez Majano S., Rachet B., Morris M. (2018). Can we assess Cancer Waiting Time targets with cancer survival? A population-based study of individually linked data from the National Cancer Waiting Times monitoring dataset in England, 2009–2013. PLoS ONE.

[11] Evans S.M., Earnest A., Bower W., Senthuren M., McLaughlin P., Stirling R. (2016). Timeliness of lung cancer care in Victoria: A retrospective cohort study. Med. J. Aust..

[12] Forrest L.F., Adams J., Rubin G., White M. (2015). The role of receipt and timeliness of treatment in socioeconomic inequalities in lung cancer survival: Population-based, data-linkage study. Thorax.

[13] Khorana A.A., Tullio K., Elson P., Pennell N.A., Grobmyer S.R., Kalady M.F., Raymond D., Abraham J., Klein E.A., Walsh R.M. (2019). Time to initial cancer treatment in the United States and association with survival over time: An observational study. PLoS ONE.

[14] Nadpara P., Madhavan S.S., Tworek C. (2015). Guideline-concordant timely lung cancer care and prognosis among elderly patients in the United States: A population-based study. Cancer Epidemiol..

[15] Vinod S.K., Chandra A., Berthelsen A., Descallar J. (2017). Does timeliness of care in Non-Small Cell Lung Cancer impact on survival?. Lung Cancer.

[16] Hall H., Tocock A., Burdett S., Fisher D., Ricketts W.M., Robson J., Round T., Gorolay S., MacArthur E., Chung D. (2022). Association between time-to-treatment and outcomes in non-small cell lung cancer: A systematic review. Thorax.

[17] Tsai C.H., Kung P.T., Kuo W.Y., Tsai W.C. (2020). Effect of time interval from diagnosis to treatment for non-small cell lung cancer on survival: A national cohort study in Taiwan. BMJ Open.

[18] Wah W., Stirling R.G., Ahern S., Earnest A. (2021). Influence of timeliness and receipt of first treatment on geographic variation in non-small cell lung cancer mortality. Int. J. Cancer.

[19] Stirling R., Brand M., Pellegrini B., Scarborough R., McNeil J., Evans S.M., Ahern S., Earnest A., Zalcberg J., on behalf of the Victorian Lung Cancer Registry (2020). The Victorian Lung Cancer Registry Annual Report.

[20] Malalasekera A., Nahm S., Blinman P.L., Kao S.C., Dhillon H.M., Vardy J.L. (2018). How long is too long? A scoping review of health system delays in lung cancer. Eur. Respir. Rev..

[21] Ansar A., Lewis V., McDonald C.F., Liu C., Rahman M.A. (2023). Factors influencing the timeliness of care for patients with lung cancer in Bangladesh. BMC Health Serv. Res..

[22] Kim M.L., Matheson L., Garrard B., Francis M., Broad A., Malone J., Eastman P., Rogers M., Yap C.H. (2019). Use of clinical quality indicators to improve lung cancer care in a regional/rural network of health services. Aust. J. Rural Health.

[23] Diaconescu R., Lafond C., Whittom R. (2011). Treatment delays in non-small cell lung cancer and their prognostic implications. J. Thorac. Oncol..

[24] Salomaa E.-R., Sällinen S., Hiekkanen H., Liippo K. (2005). Delays in the diagnosis and treatment of lung cancer. Chest.

[25] Rudin C.M., Brambilla E., Faivre-Finn C., Sage J. (2021). Small-cell lung cancer. Nat. Rev. Dis. Primers.

[26] Latimer K.M., Mott T.F. (2015). Lung cancer: Diagnosis, treatment principles, and screening. Am. Fam. Physician.

[27] Niedzwiedz C.L., Knifton L., Robb K.A., Katikireddi S.V., Smith D.J. (2019). Depression and anxiety among people living with and beyond cancer: A growing clinical and research priority. BMC Cancer.

[28] Koo M., Zhou Y., Lyratzopoulos G. (2015). Delays in diagnosis and treatment of lung cancer: Lessons from US healthcare settings. Cancer Epidemiol..

[29] Bi Q., Goodman K.E., Kaminsky J., Lessler J. (2019). What is machine learning? A primer for the epidemiologist. Am. J. Epidemiol..

[30] Li L., Lee C.C., Zhou F.L., Molony C., Doder Z., Zalmover E., Sharma K., Juhaeri J., Wu C. (2021). Performance assessment of different machine learning approaches in predicting diabetic ketoacidosis in adults with type 1 diabetes using electronic health records data. Pharmacoepidemiol. Drug Saf..

[31] Nagaraj S.B., Sidorenkov G., van Boven J.F.M., Denig P. (2019). Predicting short- and long-term glycated haemoglobin response after insulin initiation in patients with type 2 diabetes mellitus using machine-learning algorithms. Diabetes Obes. Metab..

[32] Guan X., Du Y., Ma R., Teng N., Ou S., Zhao H., Li X. (2023). Construction of the XGBoost model for early lung cancer prediction based on metabolic indices. BMC Med. Inform. Decis. Mak..

[33] Su Z., Huang F., Yin C., Yu Y., Yu C. (2023). Clinical model of pulmonary metastasis in patients with osteosarcoma: A new multiple machine learning-based risk prediction. J. Orthop. Surg..

[34] Nunez-Benjumea F.J., Gonzalez-Garcia S., Moreno-Conde A., Riquelme-Santos J.C., Lopez-Guerra J.L. (2023). Benchmarking machine learning approaches to predict radiation-induced toxicities in lung cancer patients. Clin. Transl. Radiat. Oncol..

[35] Weiss J., Raghu V.K., Bontempi D., Christiani D.C., Mak R.H., Lu M.T., Aerts H.J. (2023). Deep learning to estimate lung disease mortality from chest radiographs. Nat. Commun..

[36] Shakir H., Aijaz B., Khan T.M.R., Hussain M. (2023). A deep learning-based cancer survival time classifier for small datasets. Comput. Biol. Med..

[37] Albaradei S., Thafar M., Alsaedi A., Van Neste C., Gojobori T., Essack M., Gao X. (2021). Machine learning and deep learning methods that use omics data for metastasis prediction. Comput. Struct. Biotechnol. J..

[38] Ren C., Zhang J., Qi M., Zhang J., Zhang Y., Song S., Sun Y., Cheng J. (2021). Machine learning based on clinico-biological features integrated (18)F-FDG PET/CT radiomics for distinguishing squamous cell carcinoma from adenocarcinoma of lung. Eur. J. Nucl. Med. Mol. Imaging.

[39] Yang Y., Xu L., Sun L., Zhang P., Farid S.S. (2022). Machine learning application in personalised lung cancer recurrence and survivability prediction. Comput. Struct. Biotechnol. J..

[40] Victoria Cancer Council and Department of Health Optimal Care Pathway for People with Lung Cancer. https://www.cancer.org.au/assets/pdf/lung-cancer-optimal-cancer-care-pathway.

[41] Lababede O., Meziane M.A. (2018). The Eighth Edition of TNM Staging of Lung Cancer: Reference Chart and Diagrams. Oncologist.

[42] Oken M.M., Creech R.H., Tormey D.C., Horton J., Davis T.E., McFadden E.T., Carbone P.P. (1982). Toxicity and response criteria of the Eastern Cooperative Oncology Group. Am. J. Clin. Oncol..

[43] Australian Bureau of Statistics (2016). Census of Population and Housing: Socio-Economic Indexes for Areas (SEIFA), Australia.

[44] Australian Bureau of Statistics Australian Statistical Geography Standard (ASGS): Volume 5—Remoteness Structure, July 2016. https://www.abs.gov.au/AUSSTATS/abs@.nsf/DetailsPage/1270.0.55.005July%202016?OpenDocument.

[45] Cerulli G. (2021). Improving econometric prediction by machine learning. Appl. Econ. Lett..

[46] Garavand A., Behmanesh A., Aslani N., Sadeghsalehi H., Ghaderzadeh M. (2023). Towards Diagnostic Aided Systems in Coronary Artery Disease Detection: A Comprehensive Multiview Survey of the State of the Art. Int. J. Intell. Syst..

[47] Garavand A., Salehnasab C., Behmanesh A., Aslani N., Zadeh A.H., Ghaderzadeh M. (2022). Efficient Model for Coronary Artery Disease Diagnosis: A Comparative Study of Several Machine Learning Algorithms. J. Healthc. Eng..

[48] Ghaderzadeh M. (2013). Clinical decision support system for early detection of prostate cancer from benign hyperplasia of prostate. Stud Health Technol Inform..

[49] Sadoughi F., Ghaderzadeh M. (2014). A hybrid particle swarm and neural network approach for detection of prostate cancer from benign hyperplasia of prostate. Stud. Health Technol. Inform..

[50] Altman D.G. (1991). Practical Statistics for Medical Research.

[51] Frosch Z.A., Hasler J., Handorf E., DuBois T., Bleicher R.J., Edelman M.J., Geynisman D.M., Hall M.J., Fang C.Y., Lynch S.M. (2023). Development of a Multilevel Model to Identify Patients at Risk for Delay in Starting Cancer Treatment. JAMA Netw. Open.

[52] Pitter J.G., Moizs M., Ezer É.S., Lukács G., Szigeti A., Repa I., Csanádi M., Rutten-van Mölken M.P., Islam K., Kaló Z. (2022). Improved survival of non-small cell lung cancer patients after introducing patient navigation: A retrospective cohort study with propensity score weighted historic control. PLoS ONE.

[53] Damsees R., Jaghbir M., Salam M., Al-Omari A., Al-Rawashdeh N. (2023). Unravelling the predictors of late cancer presentation and diagnosis in Jordan: A cross-sectional study of patients with lung and colorectal cancers. BMJ Open.

[54] Hochhegger B., Alves G.R.T., Irion K.L., Fritscher C.C., Fritscher L.G., Concatto N.H., Marchiori E. (2015). PET/CT imaging in lung cancer: Indications and findings. J. Bras. De Pneumol..

[55] Desseroit M.C., Visvikis D., Tixier F., Majdoub M., Perdrisot R., Guillevin R., Cheze Le Rest C., Hatt M. (2016). Development of a nomogram combining clinical staging with 18 F-FDG PET/CT image features in non-small-cell lung cancer stage I–III. Eur. J. Nucl. Med. Mol. Imaging.

[56] Mangiameli G., Cioffi U., Testori A. (2022). Lung cancer treatment: From tradition to innovation. Front. Oncol..

[57] Forrest L., Adams J., White M., Rubin G. (2014). Factors associated with timeliness of post-primary care referral, diagnosis and treatment for lung cancer: Population-based, data-linkage study. Br. J. Cancer.

[58] Lewis R., Pereira P., Thorlby R., Warburton W. (2020). Understanding and sustaining the health care service shifts accelerated by COVID-19. Health Found..

[59] Sutherland K., Chessman J., Zhao J., Sara G., Shetty A., Smith S., Went A., Dyson S., Levesque J.F. (2020). Impact of COVID-19 on healthcare activity in NSW, Australia. Public Health Res. Pract..

[60] Al-Quteimat O.M., Amer A.M. (2020). The impact of the COVID-19 pandemic on cancer patients. Am. J. Clin. Oncol..

[61] Wassie L.A., Tsega S.S., Melaku M.S., Aemro A. (2023). Delayed treatment initiation and its associated factors among cancer patients at Northwest Amhara referral hospital oncology units: A cross-sectional study. Int. J. Afr. Nurs. Sci..

[62] Dalton S.O., Frederiksen B.L., Jacobsen E., Steding-Jessen M., Østerlind K., Schüz J., Osler M., Johansen C. (2011). Socioeconomic position, stage of lung cancer and time between referral and diagnosis in Denmark, 2001–2008. Br. J. Cancer.

[63] Forrest L.F., Sowden S., Rubin G., White M., Adams J. (2017). Socio-economic inequalities in stage at diagnosis, and in time intervals on the lung cancer pathway from first symptom to treatment: Systematic review and meta-analysis. Thorax.

[64] Baldwin D.R. (2017). Socioeconomic position and delays in lung cancer diagnosis: Should we target the more deprived?. Thorax.

[65] De Souza J.A., Hunt B., Asirwa F.C., Adebamowo C., Lopes G. (2016). Global health equity: Cancer care outcome disparities in high-, middle-, and low-income countries. J. Clin. Oncol..

[66] World Health Organization (2020). WHO Report on Cancer: Setting Priorities, Investing Wisely and Providing Care for All.

[67] Stump T.K., Eghan N., Egleston B.L., Hamilton O., Pirollo M., Schwartz J.S., Armstrong K., Beck J.R., Meropol N.J., Wong Y.-N. (2013). Cost concerns of patients with cancer. J. Oncol. Pract..

[68] Carrera P.M., Kantarjian H.M., Blinder V.S. (2018). The financial burden and distress of patients with cancer: Understanding and stepping-up action on the financial toxicity of cancer treatment. CA A Cancer J. Clin..

[69] Dickman S.L., Himmelstein D.U., Woolhandler S. (2017). Inequality and the health-care system in the USA. Lancet.

[70] Ginzberg E. (2019). Access to health care for Hispanics. Health Policy and the Hispanic.

[71] Havlik R.J., Yancik R., Long S., Ries L., Edwards B. (1994). The National Institute on Aging and the National Cancer Institute SEER collaborative study on comorbidity and early diagnosis of cancer in the elderly. Cancer.

[72] Munjal N.K., Clark R.S.B., Simon D.W., Kochanek P.M., Horvat C.M. (2023). Interoperable and explainable machine learning models to predict morbidity and mortality in acute neurological injury in the pediatric intensive care unit: Secondary analysis of the TOPICC study. Front. Pediatr..

[73] Institute of Medicine (US) Committee on Quality of Health Care in America (2001). Crossing the Quality Chasm: A New Health System for the 21st Century.

